# H5P

**DOI:** 10.29173/jchla29589

**Published:** 2021-12-01

**Authors:** Meredith P. Fischer, Matt Rohweder, Pauline Dewan

**Affiliations:** Liaison Librarian, Wilfrid Laurier University

**Product:** H5P

**URL:** https://h5p.org/

## Purpose and Intended Users

You will be interested in learning about H5P if you want plenty of options to create web content efficiently and cost-effectively. This open-source tool for producing HTML5 content is built with reuse and variety in mind. With it, you can quickly and easily design or adapt different interactive widgets and embed them into a webpage.

H5P is a useful application for anyone who teaches online. It is a great tool for instruction librarians who would like to develop open educational resources (OERs) for teaching information literacy without having to invest large amounts of time and money. Options for interactivity keep students engaged and provide opportunities for formative assessment.

## Product Description

H5P integrates with any content management system (CMS) that supports iframes. Free H5P plugins are available for Drupal and WordPress platforms.

There are over 45 different content types available. The variety can be a little overwhelming depending on how much time you have to experiment. One tip is to select a set of core content types that you want to begin using right away. The eCampusOntario H5P Catalogue (https://h5pstudio.ecampusontario.ca/) is a practical place to get a sense of the ways academic librarians use H5P.

**Table 1:** Top 10 H5P Content Types in eCampusOntario’s H5P Catalogue

H5P Content Type	Description	# of Learning Objects (as of Sept. 1, 2021)
**Quiz (question set)**	Combine multiple choice, drag text, or true/false.	320
**Course presentation**	Add interactions, audio, or video to slides.	278
**Drag text**	Create a fill-in-the-blank task with a list of words.	222
**Interactive video**	Add interactions to videos.	216
**Drag and drop**	Create a fill-in-the-blank task with a list of words or images.	181
**Accordion**	Organize information to make it more readable.	164
**Flashcards**	Create a memory game.	143
**Dialog cards**	Create turning cards.	140
**Image hotspots**	Add videos or text to an image.	130
**Fill in the blanks**	Create a fill-in-the-blank task.	84

The most popular H5P content types in the Catalogue would be an excellent starting point for a new user.

## Platform and Cost

On h5p.org, you can create a free account and test a very small selection of content types. But these content types are among the least popular with libraries and they do not give a very useful introduction to the tool. You need to use H5P on a hosted site or set up a local implementation.

One option is to use h5p.com, which does have a 30-day free trial available. This option can be expensive, but there are tiered author-based licences available that range from about $74.00 CAD for three authors to about $13,300 CAD for 1,000 authors. Enterprise licences are available as well. This route will provide you with some technical support and usage tracking. It also provides learning management system (LMS) integration for platforms like Canvas, Brightspace, Blackboard, and Moodle.

If you work at an Ontario university or college, you have the option of using the eCampusOntario H5P Studio (https://h5pstudio.ecampusontario.ca/), which provides permanent and stable hosting. You can sign up with your institutional email account. Registration is free for those who are eligible.

Local implementation is a cost-effective option, and it allows you to implement customizations around your interface. You might, for example, create a subject taxonomy for organizing H5P objects, or choose to limit content type choices to include only those that are accessible. With local implementation, you can also set up usage tracking and LMS integration.

## Special Features

This tool stands out as an option for creating OERs.

- H5P is an intuitive product that encourages sharing and adaptation with Creative Commons licences. Users can easily assign licences to their H5P objects.
- It is easy to copy and paste preexisting learning objects or download a file to adapt and repurpose Creative Commons licence content.
- PressBooks, an OER publishing tool, has an H5P plugin. Users can integrate H5P quizzes and other activities to reinforce student learning.

## Usability

No technical or advanced coding skills are needed to use H5P. Instead, the application has a strong interface that allows users to build objects easily and with little learning curve, giving them the ability to see the product as they work. Essentially, H5P utilizes a WYSIWYG editor.

## Accessibility

Several features make H5P accessible to users with a variety of accessibility needs. However, there are certain points to keep in mind when working with H5P:

- Not all content types are accessible, and a few of them are not maintained by H5P developers, such as the personality quiz and crossword.
- H5P provides up-to-date overviews of content types so that you can see which ones are accessible (https://documentation.h5p.com/content/1290410474004879128).
  - All of eCampusOntario’s top 10 content types are accessible.
- The content types listed above adhere to current Accessibility for Ontarians with Disabilities Act (AODA) standards.
- Accessible content types function with current screen reader technology and are maintained by H5P team developers.
- While many content types have full browser support (including mobile support), check H5P.org to ensure they will work on all browsers.
- Interactive Video does not have automated captioning, you will need to do captions for any videos you create.

## Strengths

- Using H5P embed code means that you only need to update content in one place, which is a major benefit if you are reusing the same H5P object on multiple webpages.
- Users can create standalone H5P objects (a quiz) or integrate a series of H5P objects into a larger project (e.g. a textbook).
- There are a wide variety of interactive content types that encourage active learning through quizzes and games.
- It is possible to add interactions to pre-existing videos retroactively.
- Some content types can function as “containers” that incorporate other content types, such as course presentations with interactive videos embedded in them or columns that use accordions to organize information.
- H5P.org has instructions and examples for the various content types.
- H5P content is responsive and looks good on anything from a phone to a laptop to a projection screen.

## Weaknesses

- Quizzes do not provide assessment data. They are, however, useful for students who want to test their knowledge.
- Some content types have limited customization and formatting options (e.g. the accordion and the image hotspot).
- H5P is not a tool for recording videos. Users must create an MP4 file with another product before using the interactive video content type.
- It is not possible to import slide presentations from PowerPoint.

## Conclusion

H5P is a fantastic option for creating interactive web content. In addition to offering a rich selection of content types, it is scalable and makes re-use easy. There are already a wide variety of H5P learning objects with Creative Commons licences out there, which means users do not always need to start from scratch. This tool is very well-suited to library instruction.

